# Dissecting genetic trends to understand breeding practices in livestock: a maternal pig line example

**DOI:** 10.1186/s12711-021-00683-6

**Published:** 2021-11-27

**Authors:** Rostam Abdollahi-Arpanahi, Daniela Lourenco, Andres Legarra, Ignacy Misztal

**Affiliations:** 1 Department of Animal and Dairy Science, University of Georgia, Athens, GA 30602 USA; 2 UMR GenPhySE, INRA Toulouse, Castanet Tolosan, France

## Abstract

**Background:**

Understanding whether genomic selection has been effective in livestock and when the results of genomic selection became visible are essential questions which we have addressed in this paper. Three criteria were used to identify practices of breeding programs over time: (1) the point of divergence of estimated genetic trends based on pedigree-based best linear unbiased prediction (BLUP) versus single-step genomic BLUP (ssGBLUP), (2) the point of divergence of realized Mendelian sampling (RMS) trends based on BLUP and ssGBLUP, and (3) the partition of genetic trends into that contributed by genotyped and non-genotyped individuals and by males and females.

**Methods:**

We used data on 282,035 animals from a commercial maternal line of pigs, of which 32,856 were genotyped for 36,612 single nucleotide polymorphisms (SNPs) after quality control. Phenotypic data included 228,427, 101,225, and 11,444 records for birth weight, average daily gain in the nursery, and feed intake, respectively. Breeding values were predicted in a multiple-trait framework using BLUP and ssGBLUP.

**Results:**

The points of divergence of the genetic and RMS trends estimated by BLUP and ssGBLUP indicated that genomic selection effectively started in 2019. Partitioning the overall genetic trends into that for genotyped and non-genotyped individuals revealed that the contribution of genotyped animals to the overall genetic trend increased rapidly from ~ 74% in 2016 to 90% in 2019. The contribution of the female pathway to the genetic trend also increased since genomic selection was implemented in this pig population, which reflects the changes in the genotyping strategy in recent years.

**Conclusions:**

Our results show that an assessment of breeding program practices can be done based on the point of divergence of genetic and RMS trends between BLUP and ssGBLUP and based on the partitioning of the genetic trend into contributions from different selection pathways. However, it should be noted that genetic trends can diverge before the onset of genomic selection if superior animals are genotyped retroactively. For the pig population example, the results showed that genomic selection was effective in this population.

## Background

Genomic selection has revolutionized the livestock breeding industry and has doubled genetic gain in some species, such as dairy cattle [1, 2]. Other livestock breeding industries, such as for pigs, broilers, beef, etc. have also heavily invested in this technology [3]. Hence, understanding whether implementation of genomic selection has been effective in these species and when the results of genomic selection became visible, are essential questions which need to be addressed.

The genetic progress of a population is a combination of the progress in different selection pathways [4], which are under different selection pressures and that have different accuracies of selection. The impact of the implementation of genomic selection on different selection pathways can be measured by the decomposition of the overall genetic trend into that contributed by each pathway over the course of selection [5].

When a population is under genomic selection, genetic evaluation based on the conventional pedigree-based method i.e., pedigree-based best linear unbiased prediction (PBLUP), is biased because it neither considers genomic information nor takes genomic preselection into account [6–8]. Genomic preselection occurs when only animals that are selected based on genomic estimated breeding values (GEBV), and therefore on positive Mendelian sampling terms, are phenotyped [9, 10]. In this case, the breeding values predicted by PBLUP for these animals are, on average, underestimated [11], while single-step genomic BLUP (ssGBLUP) is expected to provide unbiased genetic trends [12, 13] because it combines all the information that has been used for selection decisions (pedigree, genomic data, and phenotypic records). Therefore, the point when estimated genetic trends based on PBLUP and ssGBLUP diverge indicates the starting date of effective genomic selection [13]. This is the time-point when young animals are selected based on estimates of their Mendelian sampling terms based on genomics, in addition to parent average and, perhaps, own information. If all genotyped individuals are phenotyped, then the difference between genetic trends by PBLUP and ssGBLUP depends on the corresponding prediction accuracy. In principle, GEBV are more accurate than EBV and result in better genetic progress. However, if multiple-trait selection is practiced, it is important that the genetic trends are estimated using a multiple-trait model.

The average genetic merit of an individual can be decomposed as the sum of the parent average and a Mendelian sampling term that represents the deviation of an animal’s breeding value from the average of its parents. Thus, the superiority of the selected candidates over the mean of their parents represents the extra gain compared to the previous generation, by capturing the Mendelian sampling terms. Realized Mendelian sampling (RMS) terms have zero expectation when all animals are genotyped or when genotyping is at random. When animals (e.g., young boars that are kept till they reach sexual maturity and reproduce) are selected based on estimated parent average, their RMS is also 0. However, the average RMS will not be 0 when the genotyped animals are selected based on own phenotype or progeny performance, i.e. with selective genotyping. Moreover, with selective genotyping, the average RMS will be greater with ssGBLUP than with BLUP because the latter does not account for genomic information and preselection. Consequently, a deviation of the average RMS from 0 would show selective genotyping, and a divergent trend in average estimates of RMS between ssGBLUP and BLUP would display the starting date of genomic selection.

García-Cortés et al. [14] introduced a procedure for partitioning the genetic gain into contributions from parent averages and RMS and for allocating these contributions into pre-defined “paths” (e.g., by country, gender, line, etc.), summarizing path-specific terms to quantify the contributions of different sources to the overall genetic trend. This procedure has been used to quantify the contribution of different countries to the overall genetic trend in Brown Swiss bulls [15] and to explore the impact of national selection and importation in Landrace and Large-White pigs in Croatia [16].

Partitioning the total genetic trend into contributions from genotyped and non-genotyped animals or into contributions from males and females can be used to determine the impact of different selection pathways over time. For instance, if genotyped animals have a greater contribution to genetic gain than non-genotyped individuals, it can indicate selective genotyping (elite animals are genotyped) or that genomic selection is effective and most of the parents are selected from the genotyped candidates. In species such as pigs, the number of progeny is smaller per male and larger per female than in dairy cattle. Therefore, the impact of female paths on genetic progress is potentially higher than that of male paths [17] and it is worth studying this in different species.

In general, PBLUP and ssGBLUP are expected to estimate similar genetic trends before the starting date of genomic selection. However, when the elite animals are genotyped retroactively or genotyping is done after selection, PBLUP and ssGBLUP can estimate different trends. The pig population that was used here has experienced both situations for some of the traits. Thus, we used three approaches to identify and investigate changes in breeding practices over time, in particular the use of genomic selection, namely: (1) based on differences in genetic trends estimated using PBLUP versus ssGBLUP, (2) based on differences in trends in RMS estimated using PBLUP versus ssGBLUP, and (3) based on partitioning the estimated genetic trends into different selection pathways as in [14]. These approaches were applied to a real dataset from a purebred maternal pig line.

## Methods

### Data structure

The phenotypes of the Landrace pigs used in this study were collected from 2012 to 2021 and included 228,427 records for birth weight (BW), 101,225 records for average daily gain from birth to the end of the nursery period (ADG) at 11 weeks of age, and 11,444 records for average daily feed intake during the finishing period (FEED) at 23 weeks of age. FEED was measured on males only. Descriptive statistics for each trait are in Table 1. Pedigree information was available for 282,035 animals, of which 32,856 were genotyped for 36,612 single nucleotide polymorphisms (SNPs) after quality control. The animals in the pedigree were the progeny of 809 sires and 14,674 dams. The number of pigs that had either both or one parent unknown was 2011 and 835, respectively. The ratio of genotyped pigs to all pigs born in each year ranged from 4% in 2015 to 18% in 2019. Of the genotyped pigs, 92% had own records and 32% of those had offspring.

**Table 1:** Descriptive statistics of the Landrace pig breed dataset

Trait (unit)	Number of records	Mean	SD	h^2^	Minimum	Maximum
BW (g)	228,427	1281.6	327.8	0.05	45	3992
ADG (g)	101,255	343.5	61.5	0.22	200	600
FEED (g)	11,444	1917.8	301.9	0.26	827	3872

*BW* Birth weight, *ADG* average daily gain through the end of the nursery, *FEED* feed intake

### Statistical analysis

Analysis of the data was based on a multi-trait mixed linear model of the three traits considered, with the statistical model for each trait described in the following and with fixed effects denoted in uppercase and random effects in lowercase letters.

For BW, the model was:

1 $${y}_{ijokqn}={S}_{i}+{P}_{j}+{YHM}_{o}+{b(tnb}_{k})+{l}_{q}+{a}_{n}+{m}_{n}+{e}_{ijkoqn},$$

where ${y}_{ijokqn}$ denotes the BW record of animal $n$, ${S}_{i}$ is the effect of the $i$-th sex ($i$ = 1 or 2), ${P}_{j}$ is the effect of the $j$-th parity ($j$ = 1, …,9), ${YHM}_{o}$ is the effect of the $o$-th herd-year-month ($o$ = 1,…, 173), $b$ is the linear regression of BW on total number of piglets born (${tnb}_{k}$), ${l}_{q}$ is the random litter effect of sow $q$ ($q$ = 1,…, 18,394), ${a}_{n}$ is the random direct genetic effect of animal $n$ ($n$ = 1, …, 282,035), ${m}_{n}$ is the maternal genetic effect associated with dam of animal $n$, and ${e}_{ijkoqn}$ is the residual effect for the BW record.

For ADG, the model was:

2 $${y}_{ijqn}={S}_{i}+{YHW}_{j}+{l}_{q}+{a}_{n}+{e}_{ijqn},$$

where ${y}_{ijqn}$ denotes the ADG record of animal $n$, ${S}_{i}$, is the effect of the $i$-th sex ($i$ = 1 or 2), ${YHW}_{j}$ is the effect of the $j$-th year-herd-week ($j$ = 1,…, 1059), ${l}_{q}$ is the random litter effect of sow $q$ ($q$ = 1,…, 18,394), ${a}_{n}$ is the random direct genetic effect of animal $n$ ($n$ = 1, …, 282,035), and ${e}_{ijqn}$ is the residual effect for the ADG record.

For FEED, the model was:

3 $${y}_{ikqon}={B}_{i}+b\left({Age}_{k}\right)+{l}_{q}+{p}_{o}+{a}_{n}+{e}_{ikqon},$$

where ${y}_{ikqon}$ denotes the FEED record of animal $n$, ${B}_{i}$ is the fixed effect of barn $i$ ($i$ = 1,…, 512), $b$ is the linear regression of FEED on age of weighing (${Age}_{k}$), ${l}_{q}$ is the random litter effect of sow $q$ ($q$ = 1,…, 18,394), ${p}_{o}$ is the random pen effect ($o$ = 1,…, 4197), ${a}_{n}$ is the random direct genetic effect of animal $n$ ($n$ = 1, …, 282,035), and ${e}_{ijkoqn}$ is the residual effect for the FEED record.

In matrix notation, the general model for each trait can be written as:

4 $${\mathbf{y}}_{t}={\mathbf{X}\mathbf{b}}_{t}+{\mathbf{W}}_{1}{\mathbf{l}}_{t}+{\mathbf{W}}_{2}{\mathbf{p}\mathbf{e}}_{t}+{\mathbf{W}}_{3}{\mathbf{a}}_{t}+{\mathbf{W}}_{4}{\mathbf{m}}_{t}+{\mathbf{e}}_{t},$$

where ${\mathbf{y}}_{t}$ is the vector of observations for trait $t$; $t$ refers to BW, ADG, and FEED; ${\mathbf{b}}_{t}$ is the vector of fixed effects; ${\mathbf{l}}_{t}$, ${\mathbf{p}\mathbf{e}}_{t}$, ${\mathbf{a}}_{t}$, and ${\mathbf{m}}_{t}$ are the vectors of random effects for litter, pen, direct additive genetic, and maternal genetic effects, respectively; ${\mathbf{e}}_{t}$ is the vector of residuals; and $\mathbf{X}$, ${\mathbf{W}}_{1}$, ${\mathbf{W}}_{2}$, ${\mathbf{W}}_{3}$, and ${\mathbf{W}}_{4}$ are design matrices for the effects in ${\mathbf{l}}_{t}$, ${\mathbf{p}\mathbf{e}}_{t}$, ${\mathbf{a}}_{t}$, and ${\mathbf{m}}_{t}$, respectively.

The assumed (co)variance structure of random effects for the multiple-trait analysis was as follows:

5 $$\mathrm{Var}\left(\begin{array}{c}{\mathbf{l}}_{t}\\ {\mathbf{p}\mathbf{e}}_{t}\\ {\mathbf{a}}_{t}\\ \begin{array}{c}{\mathbf{m}}_{t}\\ {\mathbf{e}}_{t}\end{array}\end{array}\right)=\mathrm{Var}\left(\begin{array}{c}{\mathbf{l}}_{\mathrm{BW}}\\ {\mathbf{l}}_{\mathrm{ADG}}\\ {\mathbf{l}}_{\mathrm{FEED}}\\ {\mathbf{p}\mathbf{e}}_{\mathrm{FEED}}\\ {\mathbf{a}}_{\mathrm{BW}}\\ \begin{array}{c}{\mathbf{a}}_{\mathrm{ADG}}\\ {\mathbf{a}}_{\mathrm{FEED}}\end{array}\\ \begin{array}{c}{\mathbf{m}}_{\mathrm{BW}}\\ \mathbf{e}\end{array}\end{array}\right)=\left(\begin{array}{c}\begin{array}{ccccccccc}\mathbf{I}{\sigma }_{{\mathrm{l}}_{\mathrm{BW}}}^{2}& \mathbf{I}{\sigma }_{{\mathrm{l}}_{\mathrm{BW},\mathrm{ADG}}}&\mathbf{I}{\sigma }_{{\mathrm{l}}_{\mathrm{BW},\mathrm{FEED}}}&{\mathbf{0}}&{\mathbf{0}}&{\mathbf{0}}&{\mathbf{0}}&{\mathbf{0}}& \\ &\mathbf{I}{\sigma }_{{\mathrm{l}}_{\mathrm{ADG}}}^{2}& \mathbf{I}{\sigma }_{{\mathrm{l}}_{\mathrm{ADG},\mathrm{FEED}}}&{\mathbf{0}}& {\mathbf{0}}& {\mathbf{0}}& {\mathbf{0}}& {\mathbf{0}}& \\ & & \mathbf{I}{\sigma }_{{\mathrm{l}}_{\mathrm{FEED}}}^{2}& {\mathbf{0}}& {\mathbf{0}}& {\mathbf{0}}& {\mathbf{0}}& {\mathbf{0}}& \\ & & & \mathbf{I}{\sigma }_{{\mathrm{pe}}_{\mathrm{FEED}}}^{2}& {\mathbf{0}}& {\mathbf{0}}& {\mathbf{0}}& {\mathbf{0}}& \\ & & & & \mathbf{A}{\sigma }_{{\mathrm{a}}_{\mathrm{BW}}}^{2}& \mathbf{A}{\sigma }_{{\mathrm{a}}_{\mathrm{BW},\mathrm{ADG}}}&\mathbf{A}{\sigma }_{{\mathrm{a}}_{\mathrm{BW},\mathrm{FEED}}}&{\mathbf{A}\sigma }_{{\mathrm{a}}_{\mathrm{BW}},{m}_{BW}}& \\ & & & & & \mathbf{A}{\sigma }_{{\mathrm{a}}_{\mathrm{ADG}}}^{2}& \mathbf{A}{\sigma }_{{\mathrm{a}}_{\mathrm{ADG},\mathrm{FEED}}}&{\mathbf{0}}& \\ & & \mathrm{symmetric}& & & & \mathbf{A}{\sigma }_{{a}_{\mathrm{FEED}}}^{2}& {\mathbf{0}}&\\ & & & & & & & \mathbf{A}{\sigma }_{{\mathrm{m}}_{\mathrm{BW}}}^{2}& \\ & & & & & & & & \mathbf{R}\otimes \mathbf{I}\end{array}\end{array}\right),$$

where ${\sigma }_{\mathrm{i}}^{2}$ is the variance of the $\mathrm{i}$-th random effect; ${\sigma }_{{\mathrm{i}}_{\mathrm{j}}}$ denotes the covariance components of the $\mathrm{i}$-th effect for the $\mathrm{j}$-th combination of traits, and $\mathbf{R}$ = 3 × 3 matrix with (co)variance between traits; $\mathbf{A}$ is the numerator relationship matrix constructed based on pedigree information for PBLUP, and $\mathbf{I}$ is an identity matrix. For the ssGBLUP analysis, $\mathbf{A}$ was replaced by $\mathbf{H}$, with ${\mathbf{H}}^{-1}$ computed as in Aguilar et al. [8]:

$${\mathbf{H}}^{-1}={\mathbf{A}}^{-1}+\left[\begin{array}{cc}{\mathbf{0}}& {\mathbf{0}}\\ {\mathbf{0}}&{\mathbf{G}}^{-1}-{\mathbf{A}}_{22}^{-1}\end{array}\right],$$

where ${\mathbf{G}}^{-1}$ is the inverse of the genomic relationship matrix and ${\mathbf{A}}_{22}^{-1}$ is the inverse of the pedigree relationship matrix for genotyped individuals. The genomic relationship matrix ($\mathbf{G}$) was constructed using the first method of VanRaden [18]:

$$\mathbf{G}=\frac{\mathbf{Z}{\mathbf{Z}}^{{\prime}}}{2\sum {p}_{i} \left(1-{p}_{i}\right)},$$

where $\mathbf{Z}$ is a matrix of genotypes coded as 0, 1, and 2 for AA, AB, and BB, respectively, and then centered by subtracting twice the frequency of the major allele of SNP $i$ $({p}_{i}$) ($i$ = 1, …, 36,612). To avoid singularity problems, $\mathbf{G}$ was blended with ${\mathbf{A}}_{22}$ as $\mathbf{G}$ = 0.95 $\mathbf{G}$ + 0.05 ${\mathbf{A}}_{22}$.

Solutions for the multi-trait PBLUP and ssGBLUP were obtained using the preconditioned conjugate gradient algorithm with iteration on data, as implemented in the BLUP90IOD2 program [19]. The (co)variance components were the most recent estimates derived using PBLUP. The GEBV from ssGBLUP were set to the same base (i.e., year 2015) as the mean EBV from PBLUP. To facilitate comparisons between traits, G(EBV) were divided by the square root of the additive genetic variance.

### Criteria to dissect genetic trends

#### Divergence of genetic trends

The point of divergence of the genetic trends obtained by ssGBLUP and PBLUP was used to identify the onset of genomic selection. Details on the theory of predicting breeding values by PBLUP and ssGBLUP, are in Abdollahi-Arpanahi et al. [13]. To estimate the genetic trends using PBLUP and ssGBLUP, the (G)EBV for a given trait were averaged by year of birth for animals with both phenotypes and genotypes. The reason for using only animals with genotypes and phenotypes to estimate genetic trends is that young animals without genotypes and phenotypes (own and progeny) do not contribute information to the evaluation and their average EBV is equal to the parent average.

#### Realized Mendelian sampling terms

The RMS for individual $i$ for a given trait was estimated as:

6 $${RMS}_{i}={\left(G\right)EBV}_{i} -{PA}_{i},$$

where $PA$ is the parent average (average (G)EBV of the parents) and ${\left(G\right)EBV}_{i}$ denotes the (genomic) estimated breeding value of individual $i$. More theoretical details about the RMS can be found in Abdollahi-Arpanahi et al. [13].

When animals are randomly sampled for genotyping at a young age before any source of information (not even genomics) is available, RMS is 0 on average. However, if the “best” animals based on progeny testing or own performance are genotyped (i.e. selective genotyping), then the RMS of the genotyped animals will be nonzero, (i.e. positive if they selected for a higher value and negative if they are selected for a lower value). Since genomic preselection has taken place in most of the livestock populations, the divergence in RMS trends obtained based on EBV and GEBV of genotyped animals can also indicate the starting point of genomic selection. The same animals as used to estimate genetic trends were also used to estimate RMS trends.

#### Partitioning of genetic trends

Predictions of breeding values can be partitioned to quantify the contribution of genotyped versus non-genotyped or males versus females as follows:

7 $${\widehat{a}}_{i}=\frac{1}{2}{\widehat{a}}_{s(i)}+\frac{1}{2}{\widehat{a}}_{d(i)}+{\widehat{m}}_{i},$$

where ${\widehat{m}}_{i}$ is the estimate of the RMS and $\widehat{a}$ is the (G)EBV; subscripts $s$ and $d$ refer to the sire and dam of animal $i$, respectively. for founder animals ${\widehat{a}}_{i}={\widehat{m}}_{i}$. For the whole population Eq. (7) can be written as:

8 $$\widehat{\mathbf{a}}=\mathbf{T}\widehat{\mathbf{m}},$$

where $\mathbf{T}$ is a triangular matrix that relates each animal to its parents [20]. Following Eq. (8) and considering that $\widehat{\mathbf{m}}={\mathbf{T}}^{-1}\widehat{\mathbf{a}}$, the vector of (G)EBV for the entire population (Eq. (8)) can be partitioned into contributions of defined selection pathways [14] as:

9 $$\widehat{\mathbf{a}}=\mathbf{T}{\mathbf{P}}_{1}{\mathbf{T}}^{-1}\widehat{\mathbf{a}}+\mathbf{T}{\mathbf{P}}_{2}{\mathbf{T}}^{-1}\widehat{\mathbf{a}}={\widehat{\mathbf{a}}}_{1}+{\widehat{\mathbf{a}}}_{2},$$

where ${\mathbf{P}}_{\mathrm{i}}$ is a diagonal matrix of 1s and 0s to select the corresponding columns of $\mathbf{T}$ and is used to allocate the RMS of males versus females or of genotyped versus non-genotyped individuals to the $\mathrm{i}$-th partition of $\widehat{\mathbf{a}}$.

This procedure was implemented using the R package AlphaPart 0.8.1. [21], using the GEBV obtained using ssGBLUP for all animals (i.e., genotyped and non-genotyped) as the input. The contribution of each pathway for each birth year was expressed as a percentage by dividing the average GEBV of a partition by the average GEBV of the whole population.

## Results

### Genetic trends

**Fig. 1:**
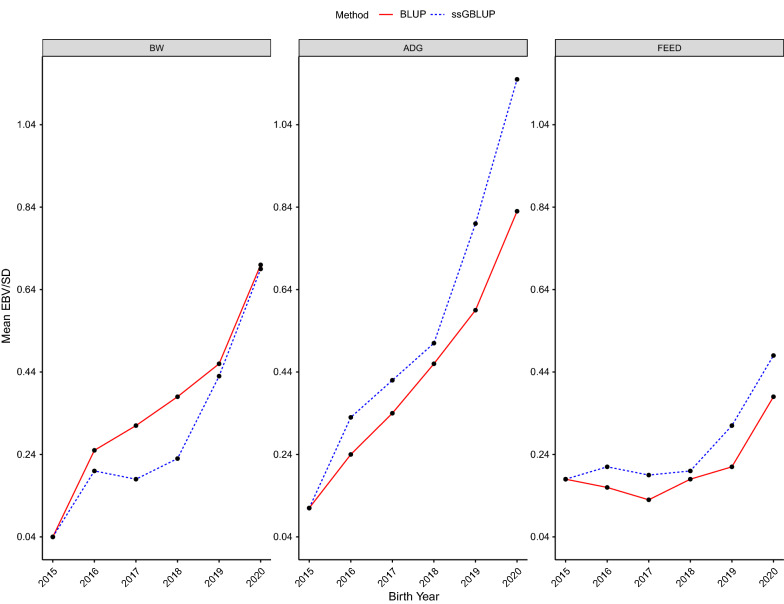
Genetic trends for birth weight (BW), average daily gain through the end of the nursery (ADG), and feed intake (FEED) for genotyped Landrace pigs. Genetic trends are presented on the additive genetic standard deviation scale and the genetic base was adjusted to the 2015 birth year

Estimated genetic trends for genotyped individuals, in genetic standard deviation units, based on PBLUP and ssGBLUP are presented in Fig. 1. The genetic trends were favorable for all traits, with a faster improvement in recent years. The changes in average EBV from 2015 to 2020 for BW, ADG, and FEED were 0.66, 0.72, and 0.20, respectively based on PBLUP and 0.65, 1.03, and 0.31 based on ssGBLUP. For ADG and FEED, the genetic trends estimated using PBLUP and ssGBLUP started to diverge in 2018, but for BW, which is not under direct selection, there was no evidence of divergence. In the last year of data (i.e., 2020), the difference between average EBV based on ssGBLUP and PBLUP were − 0.01, 0.31, and 0.11 SD for BW, ADG, and FEED, respectively. The positive genetic trend for BW is due to its indirect response to selection for increasing the maternal effect on BW and for other correlated traits in the selection index.

### Mendelian sampling trends

**Fig. 2:**
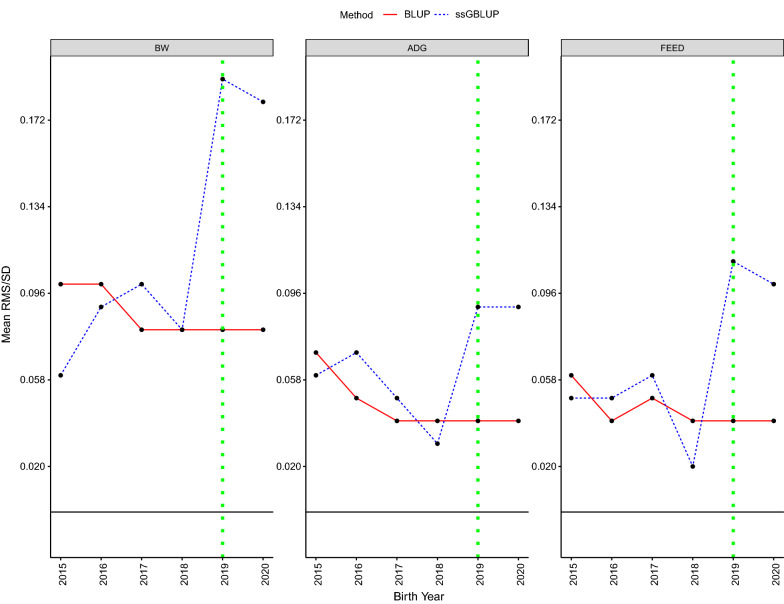
Mendelian sampling trends for birth weight (BW), average daily gain through the end of the nursery (ADG), and feed intake (FEED) for genotyped Landrace pigs. Mendelian sampling trends are presented on the additive genetic standard deviation scale. The solid black lines represent the zero-base and the dotted green vertical lines shows the start date of genomic selection

The RMS trends for genotyped individuals, in genetic standard deviation units, estimated using PBLUP and ssGBLUP are shown in Fig. 2. The pattern of changes in RMS trends was the same for the three traits and started to diverge in 2019. In the last year of data (i.e., 2020), the difference between average estimates of RMS based on PBLUP and ssGBLUP was 0.10, 0.05, and 0.06 SD for BW, ADG, and FEED, respectively. The positive estimated trend in RMS and the considerable difference of RMS from 0 from 2015 to 2017 are due to the genotyping in 2018 of elite culled or active boars that were born before 2018 and were retrieved from stored tissue samples.

### Contributions of genotyped versus non-genotyped individuals to genetic trends

**Fig. 3:**
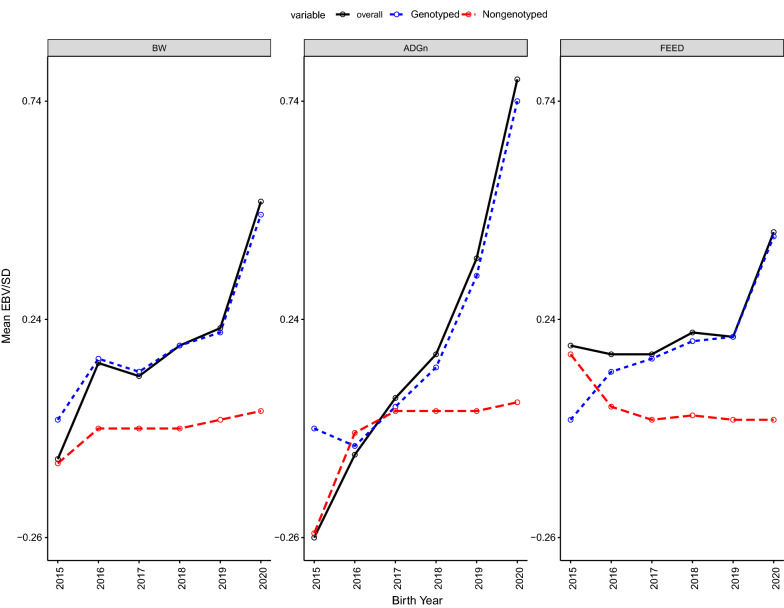
Decomposition of overall genetic trends into genotyped and non-genotyped animals for birth weight (BW), average daily gain through the end of the nursery (ADG), and average daily feed intake (FEED) for Landrace pigs. Average breeding values are presented on the additive genetic standard deviation scale

The decomposition of genetic trends into Mendelian sampling contributions from genotyped and non-genotyped individuals is shown in Fig. 3. The percentage of individuals born from 2015 to 2020 that were genotyped ranged from 5 to 21%. All genetic gain in BW was due to non-genotyped individuals before 2016, but from 2016 onwards, genotyped individuals were responsible for the genetic gain. For ADG, non-genotyped individuals had a greater contribution to the genetic trend than genotyped animals until 2016, but from 2017 onwards, the contribution of genotyped individuals to genetic gain increased from 74% in 2016 to 94% in 2020. For feed intake, in 2015, all the genetic gain was driven by non-genotyped pigs, but the contribution of genotyped pigs increased rapidly after that, from 76% in 2016 to 97% in 2020.

### Contributions of females versus males to genetic trends

**Fig. 4:**
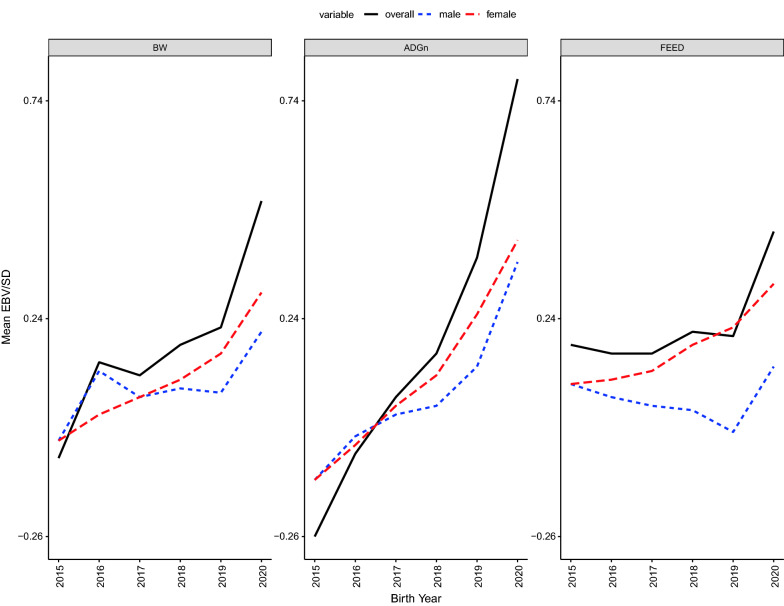
Decomposition of overall genetic trends into males and females for birth weight (BW), average daily gain through the end of the nursery (ADG), and feed intake (FEED) for Landrace pigs. Average breeding values are presented on the additive genetic standard deviation scale

Figure 4 shows the decomposition of genetic trends into contributions from males and females for all traits. For BW, most of the genetic gain was driven by females from 2019 onwards. For ADG, males and females had similar contributions to the genetic trend up to 2017, but from 2018 onwards, the contribution of females was greater than that of males. The pattern of genetic trend for feed intake was similar to that of males, while the contribution of females to the genetic trend for feed intake was in the undesirable direction. For BW and ADG, the impact of the female pathway increased after implementation of genomic selection.

## Discussion

### Genetic trends

We used the divergence of genetic and RMS trends obtained by PBLUP versus ssGBLUP to determine the effectiveness of genomic selection. We also partitioned genetic trends into contributions from genotyped versus non-genotyped animals and from males versus females. Quantifying the contribution of genotyped individuals to the genetic trends revealed the presence of selective genotyping (only elite animals were genotyped) and the impact of genomic selection (faster genetic improvement) in this breeding program. Decomposition of genetic trends into contributions from males versus females allowed the monitoring of the contribution of males versus females to genetic trends after the implementation of genomic selection.

The effective starting point of genomic selection was found to be in 2019 in the offspring of the first animals selected based on GEBV in 2018. This finding agrees with the history of genomic selection in this pig population. Genotyping started in late 2016, for pigs born between 2015 and 2017 that were active boars or culled boars with stored tissue samples. Consequently, the higher genetic trends estimated from 2015 to 2018 for ADG using ssGBLUP than using PBLUP can be the result of selective genotyping. The decline in genetic trend for ADG estimated by PBLUP after 2018 is due to genomic preselection bias. When only genotyped animals receive phenotype records, PBLUP do not account for the positive RMS of those young animals. Similar results are reported in the literature [12, 13, 22]. Masuda et al. [12] compared genetic trends estimated using PBLUP and ssGBLUP for milk production traits in US Holstein cattle and found that after the implementation of genomic selection, the genetic trend based on PBLUP was underestimated because of genomic preselection. In a simulation study, Jibrila et al. [22] showed that genomic preselection caused bias in estimates of genetic gain based on PBLUB, while the bias was smaller when based on ssGBLUP. According to Abdollahi-Arpanahi et al. [13], after implementing genomic selection in pig, broiler, and beef cattle populations, the genetic trends obtained by ssGBLUP accelerated and those estimated using PBLUP decelerated.

In the pig population under study, genotyping was done retroactively, which means that for the genotyped animals born from 2015 to 2017 the selection decisions were practiced by another method such as PBLUP, thus even if ssGBLUP during this period results in higher accuracy than PBLUP, we do not expect a higher genetic trend for ssGBLUP. In fact, the accuracy of ssGBLUP will be greater than the accuracy of PBLUP at any point when a sufficient number of animals is genotyped. However, the prediction accuracy at the time of selection is what is reflected in the genetic trend. The reason is that if the company/breeder invested in genotyping but has not used the genomic information in selection decisions, the higher accuracy of evaluation by ssGBLUP compared to PBLUP using accumulated data does not necessarily translate into the genetic trend. Overall, the changes of prediction accuracies over time may not follow the genetic trends estimated by PBLUP or ssGBLUP.

Although investigating the fluctuations in the genetic trend across time for each trait is beyond the scope of this study, the changes in genetic trends observed are consistent with the breeding practices and the periodic modifications of weights in the selection index and the genetic correlations between traits under selection. One example is the estimated increase of 0.6 and 0.7 SD in BW from 2015 to 2020 based on ssGBLUP and PBLUP, respectively. Although the direct genetic value of BW has not been selected for in this population, the maternal genetic value for BW has. The genetic correlation between direct and maternal genetic values is about 0.1. However, the genetic correlations of direct BW with growth rate in the nursery, finisher average daily gain, and finisher average daily feed intake are positive and high, e.g., 0.42, 0.31 and 0.25, respectively. Therefore, we believe that the observed genetic trend for BW is due to the correlated responses to selection.

### Mendelian sampling trends

The RMS trends revealed signatures of selective genotyping for the three traits. All males born from 2017 to 2020 were genotyped, but only a subset of females selected based on phenotypes were genotyped during this period. Hence, the deviations of RMS from 0 for birth years after 2018 are due to the strong selective genotyping of females rather than of males. Moreover, inferior males, e.g., those with a small BW, may be removed from the tested population before genotyping, which can result in positive RMS. As genotyping becomes less expensive, genotyping more young animals becomes economically justified and we expect a convergence of the RMS trends estimated using PBLUP versus ssGBLUP if phenotypic records are available for all animals.

The advantages of ssGBLUP in reducing prediction bias increase when animals have been preselected based on GEBV. Genetic evaluation using PBLUP assumes that RMS average 0, but when genotyped animals with positive or negative RMS receive phenotypes or progeny, the average RMS is no longer 0 [7, 9]. In this regard, a simulation study showed that the RMS for bulls clearly deviated from 0 after genomic preselection was implemented in a dairy cattle population [10].

### Decomposition of genetic trends

To quantify the contribution of genotyped individuals to genetic trends, we partitioned the genetic trends into the genetic gain derived by genotyped individuals and that achieved by non-genotyped individuals. Regardless of the trait, in recent years, genotyped individuals had a greater contribution to genetic gain than non-genotyped individuals. The greater contribution of genotyped individuals to genetic trends does not necessarily depict the effectiveness of genomic selection. For instance, if genotyped animals are preselected based on PBLUP EBV, we expect genetic trends to be higher for genotyped than for non-genotyped animals, which is the case for the period from 2015 to 2018 before genomic selection started.

A greater impact of females on genetic trends would be because selection decisions in a maternal line are placed more on females than males, and in pigs, each selected female has a larger contribution because it produces more progeny. However, the pig breed analyzed here is a maternal line and 40% of the traits under selection are only measured in females, which results in the females being the main drivers of changes in these traits. Thus, it is expected that females contribute more to the next generation than males in a pig breeding program, although the selection intensity for males is higher than for females. We found that the contribution of females to the genetic trends for BW and ADG was greater than that of males after the implementation of genomic selection.

For FEED, while a flat to slightly positive genetic trend was observed for males, the trend for females was positive and unfavorable. Feed intake has a negative economic value, while ADG has a positive value in the index. Therefore, the breeding objective is to achieve a positive response in growth rate and a flat or slightly positive response in FEED, which, in turn, improves feed efficiency. Few studies investigated the contribution of different selection paths to genetic trends. For example, García-Ruiz et al. [2] demonstrated that 73 to 90% of the selection differential for milk production traits in US Holstein cattle is due to the sire of the bull and sire of the cow pathways.

## Conclusions

Divergence of genetic trends for genotyped animals estimated using PBLUP versus ssGBLUP indicates the presence of genomic selection. This divergence may occur before the onset of genomic selection if superior animals are genotyped retroactively. Presence of nonzero average RMS by ssGBLUP or PBLUP indicates selective genotyping. Selective genotyping can be deliberate, e.g., genotyping of animals with superior genotypes, or incidental due to removal of weak/sick/dead animals. Under genomic selection, trends for RMS are higher when estimated using ssGBLUP than using PBLUP, with the point of divergence indicating the effective onset of genomic selection. Partitioning of genetic trends into contributions by various classes of animals such as genotyped versus ungenotyped or males versus females allows the determination of the relative impact of genotyping for different groups of animals. In particular, the observation that nearly all the genetic progress is contributed by genotyped animals confirms the increasing interest in genotyping animals, and the observation that a large fraction of the genetic progress is contributed by females validates the importance of the females in the genetic progress of a pig population. In summary, post-processing of EBV and GEBV can help to investigate the effectiveness of genomic selection and assess breeding program practices.

## Data Availability

Provider of data does not intend to disclose its identity.
